# Seizures Provoked by Greasy Pork in a Patient With Refractory Focal Epilepsy

**DOI:** 10.7759/cureus.40155

**Published:** 2023-06-08

**Authors:** Zacharia Shebani, Alexander J Brown, Camila Narvaez Caicedo, Schweta Rane, Todd Masel

**Affiliations:** 1 Neurology, The University of Texas Medical Branch, Galveston, USA

**Keywords:** video-eeg, pork-provoked seizures, seizure triggers, provoked seizures, eating epilepsy, partial seizures, semiology, seizures, epilepsy

## Abstract

Seizures have been reported to be directly triggered by certain foods in some people with epilepsy. On the other hand, eating epilepsy has been mentioned in the literature as a rare disorder characterized by clinical and EEG findings that vary from patient to patient and are interestingly prevalent in certain geographic areas. Epilepsy in these patients is either idiopathic or due to underlying brain pathology. We present a case of refractory focal epilepsy in which the patient reports seizures provoked by eating greasy pork. During the admission to the epilepsy monitoring unit (EMU), the patient did not have seizures during the first three days of the admission despite antiepileptic medication withdrawal, sleep deprivation, and photic stimulation. However, when he consumed greasy pork, he had tonic-clonic convulsions about five hours after eating. On the following day, he had another tonic-clonic seizure after eating greasy pork.

## Introduction

Eating epilepsy is a rare disorder characterized by reflex seizures triggered by food intake. Common findings, however, do arise from the reported literature [1]. Fifty-two distinct papers were recognized detailing seizure descriptions and diagnostic findings in 378 patients [1]. In these cases, eating seizures started in the second decade of life, with a greater preponderance in males [1]. They were characteristically focal-onset and most frequently in the focal impaired awareness category [1]. Medical treatment with one or several medications was observed in 80% of cases, with inadequate control reported in about 25% of patients [1]. Seizures within this category are rare, with an approximate prevalence of 0.05-0.1% within the whole epileptic population [1]. A great percentage of these cases have been identified in South Asia, signifying that eating epilepsy may be pathologically correlated to genetic or ethnic causes such as food or eating habits [1]. Although greatly stereotypical within the same patient, interindividual features can be largely diverse, with clinical signs and ictal electroencephalographic (EEG) semiology varying among patients [1]. Many of the features mentioned above are consistent with our patient, who is a male of South Asian ethnicity with underlying brain pathology and refractory focal impaired awareness epilepsy that started in the second decade of his life.

## Case presentation

A 66-year-old right-handed Cambodian male presented with a history of hypertension, hyperlipidemia, and benign prostatic hyperplasia. He endorsed seizures beginning at the age of 14, shortly after a head injury related to a bicycle wreck. He had two to three seizures until the age of 20, when they became more frequent. The patient was first started on phenytoin about 10 years after the seizure onset, and then the medication was continued for nine years with poor control (one seizure per week). Carbamazepine was started after he had migrated to the United States. It was taken with topiramate. Then the patient was tested for Human Leukocyte Antigen (HLA) genotype, given his Asian ethnicity, and found to be HLA-B*15:02 positive, indicating carbamazepine hypersensitivity. Therefore, carbamazepine was tapered to be replaced by levetiracetam. The patient reported mood changes after levetiracetam initiation. Therefore, levetiracetam was switched to valproate. Despite compliance and therapeutic levels of medication, he had seizures approximately once every 6-12 months, although sometimes there were longer intervals between events.

Shortly before the epilepsy monitoring unit (EMU) presentation, he started cenobamate at a low dose. During this period, his seizure frequency was noticeably lower. He reported seizure triggers of stress, anxiety, focusing intently, and eating pork. When he ate a sizeable piece of greasy pork (which cannot be ham), he almost always (99% of the time) had a seizure the next day. This accounted for approximately 50-70% of his seizures, with stress being the second most common trigger. He had not noticed any other food triggers. Regarding his seizure semiology, he endorsed a preceding aura with electric-like shocks in his back as well as a difficult-to-describe sensation like being pulled into the seizure. According to the patient, he tried to resist the aura, and occasionally he had isolated auras without progressing to a seizure. His typical seizures last two to five minutes, with a sudden loss of consciousness followed by rigidity and twisting of his body. His left hand flexed upward toward his face, while his right hand and lower extremities were fully extended. According to the patient, his eyes fluttered upward, and his head turned to the left. After such episodes, the patient reported going through brief confusion and experiencing extreme fatigue as if he "worked an entire day".

**Figure 1:**
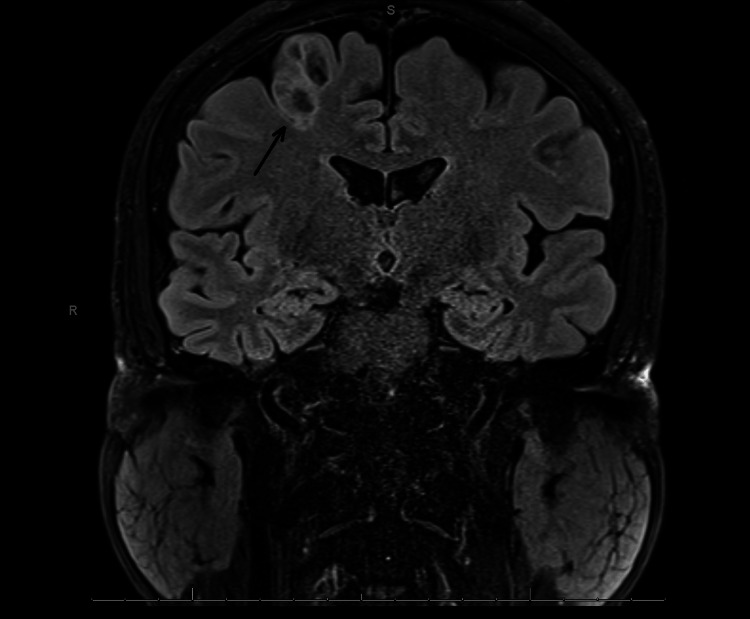
Brain MRI shows bubbly-appearing intraparenchymal cortical or subcortical lesion in the posterior aspect of the right superior frontal gyrus

**Figure 2:**
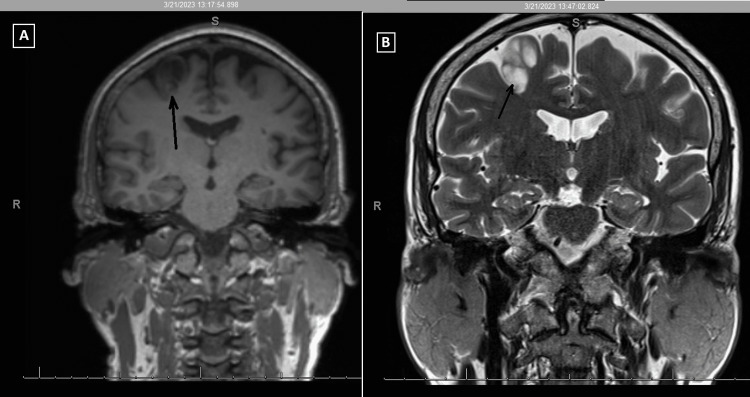
MRI of the brain. (A) T1 coronal; (B) T2/TSE coronal

He had an EEG 10 months prior to the EMU study, which reported generalized paroxysmal rhythmic high-amplitude slowing. He had an MRI one year prior, which demonstrated a posterior right frontal lobe 1.8x1.4x2.6 cm cystic non-enhancing lesion adjacent to the precentral sulcus with peripheral edema. A follow-up MRI demonstrated a non-enhancing T2-hyperintense, bubbly-appearing intraparenchymal cortical or subcortical lesion in the posterior aspect of the right superior frontal gyrus, unchanged in size or appearance from the prior MRI (Figures 1-2). The imaging findings were most characteristic of dysembryoplastic neuroepithelial tumors (DNET). Other considerations included additional low-grade CNS neoplasms such as ganglioglioma, multinodular and vacuolating neuronal tumors (MVNT), low-grade astrocytoma, or oligodendroglioma.

**Figure 3:**
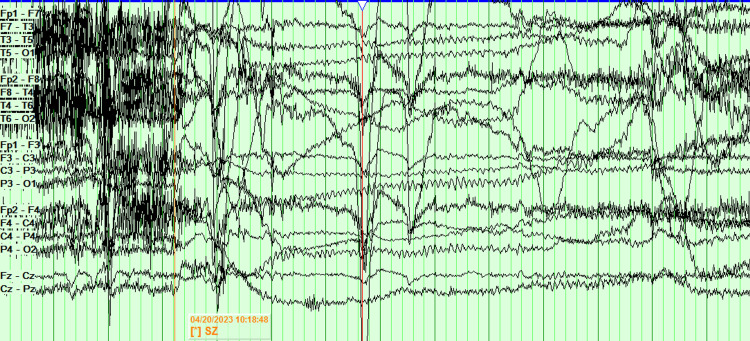
EEG of the episode is limited due to artifact. The onset of the seizure is not clear and is difficult to localize.

The patient was admitted for a five-day EMU study. On the first day, antiseizure medications were held. On the second night, the patient was sleep-deprived. On the third day, a placebo induction using verbal suggestion, along with hyperventilation and photic stimulation, while sleep deprived, failed to elicit a clinical episode or significant electrographic changes. The patient slept for several hours on the third day, ate a pork chop that evening, and was again sleep deprived on the third night. On the fourth day, the patient had two electroclinical seizures. After falling asleep on the fourth day, about five hours after eating the pork, the patient got aroused from sleep, followed by grabbing the left arm with the right hand, then head version to the left and gaze deviation to the left, along with left upper extremity flexion and right upper extremity extension. The right upper extremity then flexed and the left upper extremity extended, while the head tilted to the right while the gaze was still deviated to the left. Generalized tonic-clonic activity ensued, along with intermittent vocalization. The clinical episode lasted one minute and 13 seconds. Later that day, while sitting up and having a conversation, the patient suddenly laid down and had a similar clinical episode lasting one minute and four seconds. The scalp EEG of the seizures was limited due to excessive artifacts, and the onset of the seizure was not clear (Figure 3).

The case was then discussed in the epilepsy surgery conference, and a phase 2 intracranial EEG recording was recommended to accurately localize the seizure onset. 

## Discussion

As far as we know, this is the first case report of eating epilepsy that directly relates the occurrence of seizures strictly to greasy pork consumption (not to any other greasy food). Many of the studies that we reviewed describe cases in which seizures are related to the act of eating or drinking and speculate that the involvement of the hypothalamus in feeding could be responsible for evoking the brainstem, which in turn leads to cortical activation and the development of seizures. Our case study depreciates the role of the act of feeding or the hypothalamus because, if this hypothesis is true, the patient would have had seizures every time he consumed any food or drink, not strictly after eating greasy pork.

The exact mechanism of the relationship is unknown and has not been explained in the literature. We believe that the cause could possibly be explained by the interaction of one or more external factors (metabolic and/or digestive changes following greasy pork consumption and certain ictogenic components in the pork) and internal factors (genetic mutations and epileptogenic brain pathology). Pork is a type of red meat that is rich in protein and salt, both of which are known triggers for seizures. Fat is a known protective factor against seizures, but on the contrary, the grease seems to be potentiating the ictogenesis in this patient.

Genetics may be very strongly associated with this condition, as suggested by the predominance of eating epilepsy in Asian populations. Our patient tested positive for the HLA-B*15:02 gene, which is a gene that is found to be highly associated with an increased risk of carbamazepine-induced Stevens-Johnson syndrome and toxic epidermal necrolysis (SJS/TEN). One study concluded that a noncovalent interaction between carbamazepine and an HLA complex might lead to cytotoxic T cell-mediated cell death in patients with SJS/TEN [2]. Given that this allele and eating epilepsy are found to be predominant in Asian populations, we think of a possible correlation of the patient's condition with the HLA genotype through the possible interaction of HLA peptides with certain pork antigens, which may hypothetically lead to a series of seizure-triggering reactions. However, other responsible genetic mutations need to be sought. For instance, cases of definitive genetic causation in the pediatric group with mutations in the SYNGAP1 gene and duplication of the MECP2 gene have been reported [3]. A group of familial cases with eating epilepsy have also been documented by Senanayake [4] and Yacubian et al. [5].

The robust relation between food consumption and the following seizure event adjusts the principle of "spontaneous" ictogenesis and favors the concept of non-random seizure development associated with external modifiers of brain excitability [6]. In our patient, the brain excitability is probably caused by the brain tumor, and the epileptogenicity is then enhanced by the consumption of greasy pork by causing alterations of the baseline seizure threshold, thus making the nervous system more susceptible to initiating epileptic seizures.

The location of the epileptogenic zone varies greatly among the cases of eating epilepsy described in the literature; therefore, it does not seem to be of significant value in predicting the risk of eating-induced seizures. Further study is needed to answer many interesting mysteries surrounding this case, although this can be challenging due to the rarity of the condition.

## Conclusions

Pork with excess grease may provoke seizures in some patients with epilepsy. The exact mechanism is unknown, but multiple factors can play a role, including the interaction between external factors (chemical constituents of the pork, metabolic disturbance, and digestive changes) and internal factors (genetic mutations and epileptogenic brain pathology). We propose some risk factors based on comparing the patient data with the data reported in previous studies, including male gender, Asian ethnicity, brain lesion, and family history. Given the fact that the HLA-B*15:02 allele is prevalent in Asian populations, we suggest considering the correlation between the condition of the patient and the HLA phenotype. The location of the epileptogenic zone, EEG findings, or the seizure semiology have not been shown to be directly related. Further study is needed to clarify our arguments.
